# Antibody Immunity Induced by H7N9 Avian Influenza Vaccines: Evaluation Criteria, Affecting Factors, and Implications for Rational Vaccine Design

**DOI:** 10.3389/fmicb.2017.01898

**Published:** 2017-09-26

**Authors:** Zenglei Hu, Xinan Jiao, Xiufan Liu

**Affiliations:** ^1^Animal Infectious Disease Laboratory, School of Veterinary Medicine, Yangzhou University, Yangzhou, China; ^2^Jiangsu Co-innovation Center for Prevention and Control of Important Animal Infectious Diseases and Zoonoses, Yangzhou University, Yangzhou, China; ^3^Jiangsu Key Laboratory of Zoonosis, Yangzhou University, Yangzhou, China; ^4^Key Laboratory of Prevention and Control of Biological Hazard Factors (Animal Origin) for Agri-Food Safety and Quality, Ministry of Agriculture of China, Yangzhou University, Yangzhou, China

**Keywords:** antibody immunity, H7N9 vaccines, antigenic epitope, Treg, vaccine design

## Abstract

Severe H7N9 avian influenza virus (AIV) infections in humans have public health authorities around the world on high alert for the potential development of a human influenza pandemic. Currently, the newly-emerged highly pathogenic avian influenza A (H7N9) virus poses a dual challenge for public health and poultry industry. Numerous H7N9 vaccine candidates have been generated using various platforms. Immunization trials in animals and humans showed that H7N9 vaccines are apparently poorly immunogenic because they induced low hemagglutination inhibition and virus neutralizing antibody titers. However, H7N9 vaccines elicit comparable levels of total hemagglutinin (HA)-reactive IgG antibody as the seasonal influenza vaccines, suggesting H7N9 vaccines are as immunogenic as their seasonal counterparts. A large fraction of overall IgG antibody is non-neutralizing antibody and they target unrecognized epitopes outside of the traditional antigenic sites in HA. Further, the Treg epitope identified in H7 HA may at least partially contribute to regulation of antibody immunity. Here, we review the latest advances for the development of H7N9 vaccines and discuss the influence of serological criteria on evaluation of immunogenicity of H7N9 vaccines. Next, we discuss factors affecting antibody immunity induced by H7N9 vaccines, including the change in antigenic epitopes in HA and the presence of the Treg epitope. Last, we present our perspectives for the unique features of antibody immunity of H7N9 vaccines and propose some future directions to improve or modify antibody response induced by H7N9 vaccines. This perspective would provide critical implications for rational design of H7N9 vaccines for human and veterinary use.

## Introduction

Avian influenza A (H7N9) virus emerged in China in 2013, causing severe human infections with high mortality (Gao et al., 2013). Up to now, mainland China has experienced five epidemic waves of H7N9 influenza. As of 25 July, 2017, a total of 1,557 laboratory-confirmed cases of human infection with H7N9 avian influenza virus (AIV), including at least 605 deaths, have been reported to WHO (http://101.96.10.63/www.who.int/entity/influenza/human_animal_interface/Influenza_Summary_IRA_HA_interface_07_25_2017.pdf?ua$=$1). Of note, the fifth epidemic of human cases of H7N9 infection occurred earlier, spread to more areas and infected more people than in any earlier waves (Iuliano et al., 2017; Wang X. et al., 2017). H7N9 AIVs from the first four waves are low pathogenic in chickens and most viruses were isolated from healthy birds in live poultry market (LPM). However, on Feb 19, 2017, H7N9 virus with a four basic amino acid insertion in the cleavage site of the hemagglutinin (HA) was detected in two patients with H7N9 virus infection in Guangdong province (http://www.who.int/csr/don/27-february-2017-ah7n9-china/en/). Such strains fulfill the requirements for the classification as highly pathogenic avian influenza virus (HPAIV). Subsequently, more HPAIV H7N9 strains were isolated from humans, poultry and environment samples from LPM (Yang and Liu, 2017; Zhang et al., 2017) (http://www.oie.int/wahis_2/public%5C.%5Ctemp%5Creports/en_fup_0000024044_20170614_142756.pdf). Although, human-to-human transmission of H7N9 AIVs has been limited to a few of family clusters, the presence of multiple mammalian-adaption molecular markers has raised concerns about the potential of H7N9 viruses to be transmissible among humans and cause a next pandemic (Li et al., 2017).

In addition, according to the reports from World Animal Health Organization, H7N9 HPAIV has already caused nine severe outbreaks in poultry flocks in China since March, 2017, resulting in over 800,000 chickens being destroyed (http://www.oie.int/wahis_2/public%5C.%5Ctemp%5Creports/en_fup_0000024044_20170614_142756.pdf). Therefore, currently, H7N9 AIV poses a dual challenge for public health and poultry industry.

Vaccination is the most effective intervention to prevent influenza. However, no vaccines for H7N9 influenza are available for human and veterinary use. Since the first emergence of H7N9 influenza in 2013, a large number of vaccine candidates have been generated using various vaccine platforms. Most of them have been assessed in animal trials, but only a few have been examined in clinical trials in volunteers. The traditional criteria for immunogenicity evaluation are based on the classical serological tests, i.e., hemagglutination inhibition (HI) and virus neutralization (VN) assays. Immunogenicity of H7N9 vaccines are considered to be poor based on low HI and VN titers they induced. However, the latest findings suggest that an H7N9 vaccine is as immunogenic as H1N1 and H3N2 influenza vaccines in mice because they induced comparable total IgG antibody titers as measured by ELISA. Moreover, antibody immunity of H7N9 vaccines is also affected by multiple factors, e.g., the variation of the antigenic sites and the presence of putative inhibitory Treg epitope in HA. Here, we summarize the current advances in the development of H7N9 vaccines. Then we discuss the change of criteria for evaluation of H7N9 vaccine immunogenicity and discuss the factors affecting antibody immunity induced by H7N9 vaccines. Finally, we present our perspectives and propose directions for rational vaccine design or modification.

## H7N9 influenza vaccines in animal trials

Numerous H7N9 vaccine candidates have been generated using various technologies, including the traditional approaches, such as whole virus inactivation, split-virion and live attenuated vaccine, and cutting-edge technologies, such as virus-like particles (VLP), recombinant protein expression, nucleic acid vaccines and viral vector vaccines. Their immunogenicity and efficacy were assessed in different animal models.

A new and attractive vaccine platform based on synthetic self-amplifying mRNA allows generation of any influenza vaccine within 8 days once the sequence is available. The HA mRNA of H7N9 strain A/Shanghai/2/2013 encapsulated with lipid nanoparticle was immunogenic in mice, inducing high HI and VN antibody titers (Hekele et al., 2013). In addition, mRNA vaccines against H10N8 and H7N9 influenza generated a rapid and robust immune response in mice, ferrets and non-human primates (Bahl et al., 2017). However, no H7N9 mRNA vaccine candidate has been evaluated in clinical trials, and thus the lack of safety and efficacy data in humans may restrain the potential of mRNA vaccines to be used for influenza pandemic.

Another promising platform allowing rapid production of H7N9 vaccine is the VLP technology. Influenza VLPs are formed by a self-assembly process incorporating the surface proteins, the HA and neuraminidase (NA), into the matrix (M1) protein. The size and morphology of VLPs resemble those of influenza virus particles. The major advantage of VLPs is that they are noninfectious due to the absence of viral nucleic acid in the process of VLP assembly. Two vaccinations with the first H7N9 VLP vaccine induced high HI antibody titers and provided a good protection against H7N9 challenge in mice (Smith et al., 2013). Inclusion of adjuvant enhanced immunogenicity and efficacy of this VLP candidate. A similar VLP formulated with two saponin-based adjuvants was further tested in ferrets. Two doses of non-adjuvanted VLP in ferrets elicited high HI and VN titers and addition of either adjuvant significantly increased immunogenicity and protection against H7N9 challenge (Liu Y. V. et al., 2015). Additionally, a VLP-like H7N9 vaccine generated by baculovirus surface-displaying approach was immunogenic and completely protected mice from H7N9 challenge (Prabakaran et al., 2014). Multi-HA VLPs have been also generated to provide protection against different subtypes of H2, H7, and H9 simultaneously (Pushko et al., 2011). Generally, most H7N9 VLP vaccines are immunogenic in animal models and the adjuvant is usually added to provide better protection against virus replication and shedding.

Recombinant protein technology is also a promising approach for timely preparation of H7N9 vaccines without handling live viruses. Flublok seasonal influenza vaccines produced in baculovirus expression system do not contain adjuvants and thus a high dose (45 μg of HA) is needed to induce a robust antibody immunity (Cox et al., 2008). A comparative study of immunogenicity of HA antigens from different subtypes revealed that the HA proteins of H7N7 and H7N2 induced significantly lower neutralizing antibody titers in mice than did HAs of the seasonal influenza viruses (Blanchfield et al., 2014). For H7N9 HA-based vaccines, the full-length HA, HA2 subunit and HA globular head were expressed in either insect cells or *E. coli* (Pushko et al., 2015; Song et al., 2015, 2016; Cao et al., 2016b). The HA protein from a human-origin H7N9 strain expressed in insect cells formed subviral particles (SVP), while two intranasal immunization of the SVP induced minimal HI titers in mice without MPL adjuvant. The addition of MPL adjuvant dramatically increased HI antibody titers. Interestingly, non-adjuvanted SVP vaccine still provided 80% protection against H7N9 challenge (Pushko et al., 2015). Similarly, the HA globular head (HA1) also induced low HI titers even after two or three shots (Song et al., 2015, 2016). However, the HA2 subunit was immunogenic in mice without adjuvant and a single dose with adjuvant increased anti-HA2 IgG titers and protected mice from H7N9 challenge (To et al., 2015). Usage of different types of adjuvants can enhance antibody immunity. It is apparent that the HA-based subunit vaccines are poorly immunogenic based on low HI or VN titers.

The traditional approaches for engineering of inactivated H7N9 vaccines require handling of infectious live viruses grown in eggs or cell cultures followed by inactivation or virion disruption. The common practice is to generate reassortant viruses containing the HA and NA genes from the seasonal or pandemic influenza viruses and the remaining six internal genes from an highly egg-adapted strain A/PR/8/34 using reverse genetics. Numerous such reassortant viruses carrying the HA and NA genes from H7N9 strains have been generated. In general, non-adjuvanted whole-virus inactivated vaccines (WVIVs) induced antibody titers in a dose-dependent manner in mice, ferrets, guinea pigs, and macaques. High levels of HI, VN and IgG antibody and good protections from H7N9 challenge were only observed for high doses (Chu et al., 2014; Pan et al., 2014; Wong et al., 2014; Wodal et al., 2015; Seo and Kim, 2016). H7N9 split-virion and WVIVs showed distinctive patterns of immunogenicity in different animal models (Duan et al., 2014; Wong et al., 2014; Wu et al., 2014; Ou et al., 2016). A recent study showed that an inactivated H7N9 vaccine containing a replaced H3 HA transmembrane domain (TM) induced significantly higher HI titers, HA-specific IgG titers and IFN-γ production and provided improved interclade protection in mice compared to the vaccine containing the wild-type H7 HA (Wang Y. et al., 2017).

Live attenuated influenza vaccines (LAIV) have some advantages over WVIVs. LAIVs can establish mucosal immunity in the respiratory tract and induce cellular immunity. LAIVs based on both Leningrad/134/17/57 and A/Ann Arbor/6/60 master donor viruses were highly immunogenic in ferrets, mice and guinea pigs and are efficacious against lethal H7N9 challenge (Chen et al., 2014; Kong et al., 2015; Yang et al., 2015; de Jonge et al., 2016; Isakova-Sivak et al., 2017). However, the safety of H7N9 LAIVs must be evaluated sufficiently because the two surface proteins are derived from fatal H7N9 isolates. Moreover, viral-vectored H7N9 vaccines based on modified vaccinia virus, vesicular stomatitis virus, and human adenovirus were also immunogenic and efficacious in animal models (Kreijtz et al., 2015; Cao et al., 2016a; Wongthida et al., 2016). However, an H7HA-expressing vaccine based on parainfluenza virus 5 can provide full protection against H7N9 infection in mice, whereas it failed to induce any measurable antibody titers, suggesting a potential role of cellular immunity in protection (Chia et al., 2015).

In the veterinary field, poultry trials also present supporting evidence. For poultry influenza vaccines, HI antibody is usually used as a serological surrogate of protection and an HI titer above 16 is thought to be clinically protective. A Newcastle disease virus (NDV)-vectored H7 HA vaccine induced low H7-specific HI titers (13 after booster), whereas this vaccine provided 90% protection against HPAIV H7N7 challenge (Park et al., 2006). Similarly, a NDV-based vaccine expressing HA of H7N9 A/Anhui/1/2013 strain was low immunogenic in chickens, with only 20% of chickens being HI-positive and HI titers were around 10 after the first immunization. In contrast, in the same study, a NDV-based H5 vaccine induced seroconversion in 50% of chickens and HI titers were around 20 after the first vaccination (Liu Q. et al., 2015). In addition, our team has generated H7 HA-expressing vaccines based on different vectors, including NDV, herpesvirus of turkeys and chimeric avian paramyxovirus 2, and evaluated their immunogenicity in chickens. All these vaccine candidates consistently induced undetectable or very low HI antibody titers even after booster immunization (unpublished data).

## H7N9 influenza vaccines in clinical trials

Although, diverse H7N9 vaccine candidates were generated and positive results were obtained from animal trials, only a few have been investigated in clinical trials in volunteers. Only data from clinical trials for VLP, subunit, split-virion, and WVIVs were listed and discussed here (Table 1). For human influenza vaccines, an HI antibody titer above 40 is recognized as an immunological correlate corresponding to a 50% reduction in the risk of influenza virus infection. Novavax's H7N9 VLP vaccine based on an avian-origin virus had low immunogenicity in humans without adjuvant. Only 6 and 16% seroconversion were observed for 15 and 45 μg vaccine dose, respectively, and HI titers were below 10 after two doses. The addition of ISCOMATRIX adjuvant significantly increased antibody titers after the second dose (Fries et al., 2013). Similarly, subunit vaccines were also poorly immunogenic in adults, with 3 and 7% seroconversion rate as determined by HI and VN assays. The HI and VN titers were about 5, with no increase after the second dose. The inclusion of MF59 adjuvant dramatically improved immunogenicity of the vaccine (Bart et al., 2014). A clinical trial of split-virion vaccines formulated with or without MF59 adjuvant gave similar results that the non-adjuvanted vaccines were low immunogenic, whereas the MF59-adjuvanted immunogens induced a robust antibody immune response (Mulligan et al., 2014). Other clinical trials also demonstrated that all tested H7N9 vaccines in different formulations in the absence of adjuvant had poor immunogenicity in human beings (Jackson et al., 2015; Madan et al., 2016). Furthermore, clinical trials of vaccines for H7N1 and H7N7 subtypes also showed that they were poorly immunogenic in humans (Cox et al., 2009; Couch et al., 2012). In contrast, the monovalent pH1N1 vaccine and trivalent seasonal vaccine were highly immunogenic in healthy adult subjects without adjuvant, with seroconversion rate above 80% (Griffin et al., 2011; Grohskopf et al., 2013). However, a clinical trial of an H7N9 WVIV in Taiwan population showed that a dose of 15 μg HA induced a seroconversion rate of 40% and high HI titers without adjuvant. A higher dose (30 μg HA) or the inclusion of AI(OH3) adjuvant improved antibody response (Wu et al., 2017). It is obvious that induction of low HI titers is a common character for H7 subtype influenza vaccines. The addition of adjuvant did increase immunogenicity of the vaccines in humans, whereas the adjuvant is not currently used in standard seasonal influenza vaccines. Moreover, experts have raised some concerns about the use of adjuvants in influenza vaccination because of the likely association between adjuvanted influenza vaccination campaign and narcolepsy in children and young people in Europe (Nohynek et al., 2012; Partinen et al., 2012; Duan et al., 2014).

**Table 1:** Serological response induced by inactivated H7N9 vaccine candidates in adults.

**Vaccine type**	**Dose (μg HA)**	**Adjuvant (mg or unit)^a^**	**No**.	**Seroconversion rate (%)^b^**				**Antibody titer (geometric mean titer)**				**References**
				**Dose 1**		**Dose 2**		**Dose 1**		**Dose 2**		
				**HI**	**VN**	**HI**	**VN**	**HI**	**VN**	**HI**	**VN**	
Subunit	3.75	MF59 (4.875)	94	0	0	26	55	5.1	5.7	12.0	42.0	Bart et al., 2014
	7.5	MF59 (9.75)	103	0	1	44	73	5.0	6.2	19.0	76.0	
	15	MF59 (9.75)	97	0	4	52	78	5.0	7.1	26.0	100	
	15	None	102	1	1	3	7	5.1	5.3	5.8	7.7	
VLP	5	ISCO (30U)	37	0	–	65	–	5.4	–	37.1	–	Fries et al., 2013
	15	ISCO (30U)	38	3	–	37	–	5.8	–	22.7	–	
	5	ISCO (60U)	36	0	–	81	–	6.7	–	64.3	–	
	15	ISCO (60U)	34	0	–	65	–	5.4	–	37.1	–	
	15	None	35	3	–	6	–	5.6	–	6.8	–	
	45	None	32	3	–	16	–	5.7	-	9.6	–	
Split virion	3.75	MF59 (9.75)	99	3	8	59	82	6.0	11.7	33.0	81.4	Mulligan et al., 2014
	7.5	MF59 (9.75)	98	4	7	58	73	5.9	11.6	33.8	63.8	
	15	MF59 (9.75)	99	4	6	47	71	6.0	11.9	25.3	62.2	
	15	MF59 (9.75)/none^c^	98	4	8	35	66	6.1	11.7	19.6	43.5	
	15	None/MF59 (9.75)	99	1	1	20	40	5.2	6.2	10.2	25.8	
	15	None	100	1	2	6	13	5.2	6.6	6.4	11.6	
	45	None	99	2	1	5	21	5.5	8.2	6.5	16.3	
Split virion	3.75	AS03 (5.93)	56	14	8	86	92	13.4	33.8	92.9	360	Madan et al., 2016
	3.75	AS03 (11.86)	56	14	19	89	92	15.8	45.6	128	603	
	7.5	AS03 (5.93)	54	19	8	89	89	16.5	37.5	106	325	
	7.5	AS03 (11.86)	55	32	25	96	93	18.4	44.2	151	485	
	15	None	56	7	0	23	33	10.5	26.4	17.2	62.2	
Split virion	3.75	AS03 (10.68)	95	6	12	91	97	6.7	14.4	107	171	Jackson et al., 2015
	7.5	AS03 (10.68)	94	11	19	81	87	7.6	15.0	80.9	114	
	15	AS03 (10.68)	96	10	13	84	92	8.2	15.0	103	119	
	15	AS03 (10.68)/none	93	16	18	63	81	9.2	17.2	43.1	74.6	
	15	None/AS03 (10.68)	97	1	2	28	44	5.2	6.5	13.0	32.2	
	15	AS03(10.68)/MF59(9.75)	92	8	15	70	82	7.1	13.7	41.5	63.1	
	15	MF59(9.75)/AS03(10.68)	96	6	6	75	85	6.5	10.6	58.6	96.9	
	15	MF59 (9.75)	94	4	4	57	74	5.9	9.3	29.0	52.2	
	15	None	94	1	2	2	12	5.2	6.5	5.9	10.3	
	45	None	95	2	5	10	19	5.4	7.6	7.6	15.7	
Whole-virus inactivated	15	AI(OH3)(0.3)	50	4.1	0.0	37.5	31.3	9.4	5.1	21.8	17.8	Wu et al., 2017
	30	AI(OH3)(0.3)	50	10.2	10.2	64.6	41.7	12.4	7.4	36.2	25.6	
	15	None	50	11.1	2.2	40.0	26.7	11.7	6.5	24.1	17.1	
	30	None	50	20.0	10.0	46.9	38.8	15.2	7.8	32.8	23.0	

a *ISCO, ISCOMATRIX. Units for ISCOMATRIX adjuvant*.

b *HI, hemagglutination inhibition; VN, virus neutralization*.

c *Adjuvant added to one of two doses*.

## Criteria for evaluation of immunogenicity of H7N9 vaccine candidates

Although, HI and VN antibodies are well understood to correlate with protection for the seasonal influenza vaccines, it is not clear whether these serological assays also correlate with protection against avian influenza viruses such as H7 subtype. It is vital to point out that evaluation of immunogenicity of most H7N9 vaccines in animal models and humans described above was based on the traditional HI and VN tests. H7N9 vaccines are apparently low immunogenic due to low HI and VN antibody titers they induced.

However, a latest study has demonstrated that evaluation of H7N9 vaccines based on only HI and VN may result in underestimation of immunogenicity of vaccines (Kamal et al., 2017). In their study, BALB/c mice were vaccinated with non-adjuvanted inactivated antigens derived from H7N7, H7N2, H7N9, H1N1, and H3N2 viruses. H7 subtype antigens induced significantly lower HI and VN titers compared to H1 and H3 subtypes, whereas they elicited comparable high IgG antibody titers measured by ELISA. This indicates that H7 subtype antigens are as immunogenic as the seasonal influenza antigens based on overall IgG antibody response.

Technically, both HI and VN antibodies are a part of IgG antibody, while the proportion of HI and VN antibodies induced by H7 antigens in total IgG antibody is significantly lower compared to their seasonal counterparts. A large fraction of antibody has no virus-neutralizing activity *in vitro*. More importantly, naïve mice passively immunized with the H7 immune serum were partially protected against lethal H7N7 challenge (Kamal et al., 2017). This suggests a significant role of non-neutralizing antibody (non-nAb) induced by H7 antigens in protective immunity *in vivo*. Similarly, two independent studies showed that both neutralizing and non-neutralizing antibody confer protection through divergent mechanisms (Henry Dunand et al., 2016; Tan et al., 2016). These findings indicate that the apparently low immunogenicity of H7N9 vaccines is at least partly related to measuring antibody titers with the traditional HI and VN assays, which may not provide a true measure of protective immunity associated with H7 immunization. The development of additional correlates of immunogenicity of H7 vaccines is needed.

## Factors affecting antibody immunity induced by H7N9 vaccine candidates

H7N9 vaccines produced an equivalent level of overall IgG antibody with the seasonal influenza vaccines, whereas a large fraction of the antibody was not neutralizing *in vitro*. This is a remarkable signature of the quality of antibody immunity for H7N9 vaccines. Changes in the antigenic epitopes in HA may at least partially account for the production of non-nAb after H7N9 immunization.

In a recent report (Tan et al., 2016), neutralizing monoclonal antibody (nAb) against the HA protein of a human H7N9 isolate bound to the epitope homologous to the antigenic site A of H3 HA, as determined by escape mutants assays. However, non-nAbs did not show any diminished binding to nAb-generated escape mutants, suggesting that non-nAbs did not bind to site A. In addition, since non-nAbs have no HI activity, it is likely that these antibodies would bind at some distance from the receptor binding site of HA. A panel of H7 HA mutants were screened for decreased binding of non-nAbs and diminished binding was observed for a mutant carrying R65K mutation. This site is located at the interface between the HA globular head and stalk region, which may be the target epitope recognized by non-nAbs.

Similarly, Dunand et al. cloned monoclonal nAbs and non-nAbs from plasmablasts isolated from H7N9-vaccinated humans (Henry Dunand et al., 2016). A competition assay showed that non-nAbs exhibited low levels of competition with stalk-reactive nAbs, indicating that they bind to different epitopes. At low pH, the HA stalk domain undergoes conformational changes to promote membrane fusion. nAbs binding to the stalk region can prevent membrane fusion by inhibiting conformational changes. An antibody binding test using viruses treated with various pH-buffered (7.4–4.4) solutions revealed that exposure to low pH prevented binding of mAbs that recognize conformational epitopes on the pre-fusion HA. Interestingly, non-nAbs bound to HA in a post-fusion state (pH 5.0 and 4.4), suggesting that the epitope targeted was not affected by conformational changes and even become more accessible in the post-fusion state. Therefore, non-nAbs bind to epitopes outside of the classical binding sites, and these epitopes might be conformational epitopes. Although, epitopes targeted by non-nAb induced by H7N9 vaccines are still unidentified, it is clear that non-nAb plays a significant role in protection and the mechanism is the immune effects e.g. phagocytosis mediated by the interaction between IgG Fc and Fc receptors.

Another factor influencing antibody immunity for H7N9 vaccines is the presence of Treg epitope in HA. An immunoinformatics team has identified that the T cell epitopes in H7 HA are highly conserved, on the T cell receptor face, with a large number of similar epitopes in human genome (Liu R. et al., 2015). This may lead to evasion of the HA protein from the immune response. Moreover, the magnitude of human T cell effector responses to individual H7N9 T cell peptides was inversely correlated with the peptide's resemblance to the host. An inhibitory T cell epitope in HA was identified, which suppressed responses to other H7 peptides and peptides of the seasonal influenza viruses in human peripheral blood mononuclear cells (PBMC). The underlying mechanism is that this peptide in HA has a high conservation with human genome, mediating “immune camouflage” and was shown to expand CD4^+^FoxP3^+^ regulatory T cells (Treg), mediating a negative regulation of the immune response (Liu R. et al., 2015). Thus, H7N9 AIVs may evade the immune response through “immune camouflage” and activation of Treg response. The presence of the inhibitory Tregitope in H7 HA was thought to be associated with low seroconversion rates and HI antibody titers induced by H7N9 vaccines in humans. However, there is no direct and solid evidence to support this speculation.

Since HI and VN titers are not sufficient to evaluate immunogenicity of H7N9 vaccines, it is necessary to investigate the effect of Treg in HA on serum IgG antibody. Wada et al. compared immunogenicity of H7N9 and H3N2 vaccines in a humanized mouse model (Wada et al., 2017). In BALB/c mice, an inactivated H7N9 vaccine and the recombinant H7 HA protein induced comparable HA-reactive IgG antibody titers as their H3N2 counterparts. However, in humanized mice that were transplanted with healthy human PBMC, IgG titers elicited by H7N9 antigens were significantly lower than that induced by H3N2 antigens. An explanation is that PBMC donors may have an H3N2 exposure history and H3N2 immunization activated the memory immunity. Intriguingly, when certain amino acids in the Treg epitope were mutated to those in H3N2, the optimized H7 antigens induced significantly higher IgG titers in humanized mice than did the wild-type HA. It is obvious that Treg exhibited a suppressive effect on IgG antibody titers measured by ELISA induced by H7N9 antigens in humanized mice. However, the optimized H7 HA failed to increase IgG antibody in mice transplanted with H3N2-primed murine splenocytes. It is probable that immune history of animal models may affect antibody immunity against influenza viruses and the function of Treg may be human leukocyte antigen (HLA)-restricted.

A further question we asked is that whether the localization of the T cell epitopes has some relationship with their immune regulatory effects. We found that 11 predicted T cell epitopes localize in different regions in H7 HA: 5 in the globular head, 4 in the stalk domain, 1 in the signal peptide and 1 in the TM domain (Table 2). It is noted that the inhibitory Treg epitope identified in the above immunoinformatics study localizes in the stalk region, indicating that the HA stalk may play a role in the regulation of the immune response. Intriguingly, the stalk region of H7N9 HA indeed had a suppressive effect on immunogenicity of the HA head (He et al., 2016). Different regions of HA, including the globular head, the stalk and the ectodomain (contains the head and stalk), fused with the intramolecular adjuvant IgG Fc were expressed. These vaccines were formulated with MF59 adjuvant for immunization study in mice. HI and VN antibody titers induced by the stalk-, head plus stalk-, and ectodomain-based vaccines were significantly lower than those induced by the HA head alone. This suggests that immunogenicity of HA head is significantly suppressed as long as the HA stalk is present, which provides important implications for rational design of HA-based vaccines. The author also surmised that domains or epitopes that are potentially suppressive to the neutralizing immunogenicity of HA head may exist in the stalk.

**Table 2:** Location of the predicted T cell epitopes in the HA protein.

**Epitope**	**Position**	**Location in HA^b^**			
		**Head**	**Stalk**	**SP^c^**	**TM^d^**
CPRYVKQRSLLLATGMKNVPE^a^	314–334				
YAEMKWLLSNTDNAAFPQ	155–172				
RIDFHWLMLNPNDTVTFS	238–255				
RASFLRGKSMGIQSG	265–279				
DKLYERVKRQLRENAEED	455–472				
AMGLVFICVKNGNMRCT	541–557				
GFTYSGIRTNGATSSCRR	132–149				
PGKFVNEEALRQILR	107–121				
MNTQILVFALIAIIPTNADKI	1–21				
IDQITGKLNRLIEKT	384–398				
NLPFQNIDSRAVGKC	301–313				

a *The inhibitory T cell epitope identified using the immunoinformatics tools*.

b *The color values indicate the regions of HA*.

c *SP, signal peptide*.

d *TM, transmembrane domain*.

## Perspectives and future directions

Antibody response induced by H7N9 vaccines is qualitatively different from that induced by the seasonal influenza vaccines. H7N9 vaccines elicit low HI and VN titers but high overall IgG antibody titers. Thus the traditional HI and VN assays are not sufficient to evaluate immunogenicity of H7N9 vaccines. New immunological correlates, such as ELISA titers, to protection should be established. In addition, compared to the seasonal influenza vaccines, a large proportion of IgG antibody induced by H7N9 vaccines is non-nAb, indicating that epitopes recognized by non-nAb might be dominant epitopes in H7 HA. In addition, the immune history of animal models has an important impact on antibody immunity induced by influenza antigens, which should be taken into account when immunogenicity of H7N9 vaccines is evaluated. Although, there is no sufficient supporting evidence for the role of Treg, the presence of Treg epitope in H7 HA may partially account for low immunogenicity of H7N9 vaccines in a humanized mouse model and humans.

These novel findings present critical implications for H7N9 vaccine design. There are several directions to improve or modify immunogenicity of H7N9 vaccines: (1) the molecular mechanism of the induction of non-nAbs by H7N9 antigens requires further studies; (2) the epitopes targeted by non-nAb require to be identified and it may be doable to guide the host immune response toward the classical neutralizing epitopes by shielding non-nAb-recognized epitopes to enhance HI or VN antibody response; (3) the presence of Treg epitope in poultry and mammal models needs to be identified, and the mechanism of the inhibitory effect of Treg epitope on antibody response requires further understanding; (4) the inhibitory Treg epitope in HA may be related to low antibody levels in humans, it is reasonable to modify or remove this epitope or the stalk containing this epitope to enhance immunogenicity.

## Author contributions

ZH designed and drafted the manuscript. XJ and XL revised the manuscript.

### Conflict of interest statement

The authors declare that the research was conducted in the absence of any commercial or financial relationships that could be construed as a potential conflict of interest.
